# PrEP discontinuation among Latino/a and Black MSM and transgender women: A need for PrEP support services

**DOI:** 10.1371/journal.pone.0241340

**Published:** 2020-11-05

**Authors:** Omar Nieto, Ronald A. Brooks, Amanda Landrian, Alejandra Cabral, Anne E. Fehrenbacher

**Affiliations:** 1 Department of Family Medicine, University of California, Los Angeles, California, United States of America; 2 Center for HIV Identification, Prevention, and Treatment Services (CHIPTS), University of California, Los Angeles, California, United States of America; 3 Department of Community Health Sciences, Fielding School of Public Health, University of California, Los Angeles, California, United States of America; 4 Department of Psychiatry and Biobehavioral Sciences, Semel Institute for Neuroscience and Human Behavior, University of California, Los Angeles, California, United States of America; Ohio State University, UNITED STATES

## Abstract

**Purpose:**

Disparities persist in HIV infection among Black and Latino men who have sex with men (BLMSM) and Black and Latina transgender women (BLTW). Increasing uptake and subsequent consistent use of pre-exposure prophylaxis (PrEP), an effective biomedical strategy for preventing HIV acquisition, can dramatically reduce HIV incidence in these populations. The purpose of this study was to explore reasons for PrEP discontinuation among BLMSM and BLTW living in Los Angeles County to inform the development of support services for these populations to remain persistent with their PrEP regimen.

**Methods:**

In-depth, semi-structured interviews were conducted with 15 BLMSM and 7 BLTW who reported either temporary or indefinite PrEP discontinuation. A thematic analysis approach was used to analyze qualitative data.

**Results:**

Four themes emerged related to reasons for PrEP discontinuation, including: (1) lower perceived HIV risk related to changes in sexual behavior; (2) structural or logistical barriers (e.g., lapse or loss of health insurance, cost, difficulty navigating complex medical systems); (3) anticipated and experienced medication side effects, with a sub-theme of interactions between PrEP and feminizing hormone medications; and (4) challenges with medication adherence.

**Conclusions:**

PrEP is an important prevention tool for BLMSM and BLTW, particularly during periods of heightened HIV risk. However, both individual (e.g., inability to adhere to medication, changes in HIV sexual risk behaviors) and structural/logistical (e.g., loss of insurance, navigating complex medical systems) factors can cause temporary or indefinite PrEP discontinuation. Additional support services, beyond those offered by medical providers, are needed to help BLMSM and BLTW PrEP users overcome barriers to discontinuation and assist them to remain persistent with their PrEP regimen. We describe potential options for support services such as PrEP case management, expanded PrEP navigation services, or text messaging services.

## Introduction

HIV disparities persist among Black and Latino men who have sex with men (BLMSM) and Black and Latina transgender women (BLTW). The Centers for Disease Control and Prevention (CDC) estimates that one in two Black MSM and one in five Latino MSM will be infected with HIV in their lifetime, compared with one in eleven white MSM [1]. Additionally, in 2018, BLMSM accounted for the largest percentage (68%) of all new HIV diagnoses among gay and bisexual men [2]. The rates of HIV diagnosis are also pronounced among BLTW. According to the CDC, of all HIV diagnoses among transgender women (TW) from 2009 to 2014, 51% were Black TW, 29% were Latina TW, and only 11% were among white TW [3]. These alarming trends signal an urgent need to increase the uptake and continued use of effective prevention strategies, such as Pre-Exposure Prophylaxis (PrEP), among high-risk persons living at the intersections of racial, ethnic, sexual, and gender-diverse identities.

PrEP is an efficacious biomedical prevention strategy with the potential to dramatically reduce the rate of incident HIV infections in BLMSM and BLTW [4–6]. PrEP involves daily use of one of two HIV medications, Truvada® or Descovy®, to prevent HIV acquisition [7, 8]. Increasing PrEP use is a principal strategy of the Health and Human Services’ “Ending the HIV Epidemic: A Plan for America” initiative [9]. After PrEP initiation, safe and effective use depends on adherence to the medication, retention in routine PrEP medical monitoring, and consistent use during periods of high-risk behaviors. Difficulty adhering to these behaviors contributes to reduced protection against HIV infection among BLMSM and BLTW PrEP users [10–14].

BLMSM and BLTW may experience multiple structural, logistical, and individual-level challenges to using PrEP consistently. Commonly cited structural and logistical barriers include the inability to afford the medication, loss of health insurance or access issues, change in health care provider, moving out of state, and transportation issues [11, 12, 15–18]. Individual-level challenges include experiencing medication side effects or negative reactions from family and friends related to the use of PrEP for HIV prevention [11, 12, 16–18]. While these studies, primarily quantitative, provide some information on reasons for discontinuation among BLMSM [12, 17], no studies have qualitatively assessed reasons for discontinuation specifically among BLTW.

In this sub-analysis of our main study on PrEP-related stigma, we explored the reasons for PrEP discontinuation in a sub-sample of BLMSM and BLTW. Understanding the reasons for PrEP discontinuation will help inform the development of support services for these populations to remain persistent with their PrEP regimen and HIV-negative.

## Methods

### Main study overview

Between January 2017 and November 2018, a purposive sample of BLMSM and BLTW PrEP users was recruited to participate in in-depth qualitative interviews to explore their experiences of PrEP-related stigma (i.e., anticipated, enacted, internalized) [19–21]. Participants were recruited through gay-oriented sexual and social networking apps (i.e., Grindr and Growlr), a LGBTQ community agency or participant referral, and dissemination of study promotional materials at population-specific community events. Individuals were eligible to participate in the main study if they were 18 years of age or older, identified as a Latino/a or Black/African American cisgender man who has sex with men or transgender woman, had anal sex with a male partner in the previous six months, were currently or had previously taken Truvada® for PrEP (Descovy had not yet been approved by the FDA at the time of the study), and resided in Los Angeles County (LAC). Study recruitment was terminated once data saturation was reached (i.e., no new information was being gathered from interviews).

### Study procedures

A semi-structured interview guide was used to assess experiences of PrEP-related stigma. Participant PrEP use was verified with a prescription bottle prior to the initiation of the study procedures or through self-report for individuals who had discontinued PrEP prior to the first interview. Interviews were conducted in English at a university-based research clinic by one of the research team members (ON), who also matched many of the demographic characteristics of the study population (e.g., race, sexual orientation, PrEP use). Prior to recruitment, the study interviewer received training by the principal investigator (RAB) in conducting qualitative interviews. All participants were assigned a unique identification number to maintain confidentiality. Interviews were audio recorded and lasted 30 to 60 minutes. An Audio Computer-Assisted Self-Interview (ACASI) survey was also administered to all participants to gather information on demographic and PrEP use characteristics.

Follow-up interviews were conducted with BLMSM to assess changes in experiences of PrEP stigma from baseline to 6-month follow-up. With limited supplemental funding, the study population was expanded to include BLTW. However, we were unable to conduct follow-up interviews with these participants. All interview audio files were transcribed verbatim and checked for accuracy by two research assistants. The Institutional Review Board of the University of California, Los Angeles approved all study materials. All participants provided written informed consent and received a $50 gift card for their participation. BLMSM received an additional $50 gift card for completing the follow-up interview.

### Sub-analysis methods

We conducted a sub-analysis of PrEP discontinuation among participants who completed a study interview for the main study (see S1 Appendix to view the relevant study questions). Participants were included in the sub-analysis if they reported discontinuing PrEP during the initial interview or follow-up interview (only conducted with BLMSM) either temporarily (i.e., stopped using PrEP for a period but then re-initiated) or indefinitely (i.e., stopped using PrEP but may re-initiate in the future).

### Data analysis

Interview transcripts were iteratively coded, sorted, and analyzed using a thematic analysis approach [18]. Initial codes were developed from the interview guide, field notes, and multiple readings of the transcripts. The study team, comprised of the principal investigator and two research assistants, reviewed and discussed the codes and their definitions, refined and deleted codes, and identified exemplar quotes associated with each code before reaching consensus on the final codebook. A subset of these codes was selected for a test of inter-coder reliability. Two research assistants independently coded randomly selected interview transcripts and an inter-coder reliability (ICR) score was computed for baseline interviews with BLMSM (Cohen’s kappa coefficient, k = 0.92) and BLTW (k = 0.87). A separate ICR score was also computed for follow-up interviews with BLMSM (k = 0.84). Final codes were entered into ATLAS.ti (version 8.0.42) and used to code associated quotations for all transcripts. Once coding was complete, the study team reviewed the data exports and created an additional coding scheme to organize data into broad categories. These categories were then refined to identify emerging themes related to PrEP discontinuation. Themes were determined by their prominence in the overall dataset (i.e., frequency or the depth of a specific discontinuation factor being discussed across all participants) [18]. While some participants (n = 9, 40.9%) noted that multiple factors contributed to their decision to stop using PrEP, most did not identify a primary factor.

## Results

From our main study sample of 81 participants, we analyzed data from 22 participants who indicated that they had discontinued PrEP, whether temporarily or indefinitely. The discontinuation sample consisted of 15 BLMSM and 7 BLTW. Demographic and PrEP use characteristics of the sample are presented in Table 1. The mean age of participants was 32 years old. A little more than half (54.5%) reported having an annual household income of less than $10,000 and the majority were insured (90.9%) (e.g., Medicare/Medicaid, Medi-Cal, private or employer-provided insurance). The average length of time on PrEP was 15 months (standard deviation = 12.6, median = 11.5, range 2–44 months), with the average length of discontinuation at 6 months (standard deviation = 9.7, median = 3, range = 0.5–44 months).

**Table 1:** Participant demographic characteristics (N = 22).

Variable	N (%) or M, SD
***Demographic Characteristics***	
Age (in years)	M = 31.9, SD = 9.4
Race/Ethnicity and Gender Identity	
Black/African-American Cisgender Men	8 (36.4)
Latino Cisgender Men	7 (31.8)
Black/African-American Transgender Women	3 (13.6)
Latina Transgender Women	4 (18.2)
Employment status	
Employed (full or part time)	9 (40.9)
Unemployed or on temporary/permanent disability	11 (50.0)
Other	2 (9.1)
Annual income	
$0–9,999	12 (54.5)
$10,000–19,999	2 (9.1)
$20,000–39,999	6 (27.3)
$40,000–59,999	2 (9.1)
Health insurance	
Medicaid or Medicare	12 (54.6)
Medi-Cal	4 (18.2)
Private or employer-provided insurance	4 (18.2)
Insurance through a parent or partner	2 (9.1)
VA coverage	1 (4.5)
Does not have health insurance	2 (9.1)
Relationship status	
Single and not dating anyone special	14 (63.6)
Dating someone in an open relationship (i.e., have sex with other people)	3 (13.6)
Dating someone in a closed relationship (i.e., don’t have sex with other people)	3 (13.6)
Partnered/married in an open relationship (i.e., have sex with other people)	1 (4.5)
Partnered/married in a closed relationship (i.e., don’t have sex with other people)	1 (4.5)
***PrEP Use Characteristics***	
Length of time using PrEP (in months) (N = 22)	M = 14.76, SD = 12.55
Length of time PrEP discontinuation (in months) (N = 19)^1^	M = 6.09, SD = 9.73

^1^Excludes participants who did not clearly indicate the length of time they discontinued PrEP.

Four themes related to PrEP discontinuation among BLMSM and BLTW were identified. They include: (1) lower perceived HIV risk related to changes in sexual behavior; (2) structural or logistical barriers; (3) anticipated and experienced medication side effects, with a sub-theme of interactions between PrEP and feminizing hormones; and (4) challenges with medication adherence.

### Lower perceived HIV risk related to changes in sexual behaviors

A prominent factor contributing to PrEP discontinuation was a lower perceived risk of HIV infection. This typically occurred following a change in sexual risk behaviors, such as the participant deciding to use condoms consistently rather than continue with PrEP (Table 2, Quote 1). Others were motivated to stop after experiencing a decrease in sexual encounters (Quotes 2–3), with some indicating that they only used PrEP during periods of heightened sexual risk taking (e.g., “seasons of risk”). Those who experienced a change in personal life circumstances that led to a reduction in HIV risk behavior were also motivated to discontinue the medication (e.g., no longer engaging in sex work, stably housed; Quote 4). In addition, some participants no longer saw the need for PrEP once they entered a mutually monogamous relationship with their partner (Quotes 3, 5–6).

**Table 2:** Lower perceived HIV risk related to changes in sexual behavior.

1.) I just decided, “Well, I’m using condoms and I’m pretty careful about using condoms.” (Latino MSM, age 40)
2.) The reason why I stopped using PrEP is because I’m not having sex like that. (Black MSM, age 55)
3.) [I stop using PrEP] periodically. I don’t know why, but if I’m not [sexually] active… I recently stopped maybe like when the summer was starting. I’ve just been just kind of like staying to myself… I was consistent the last time we talked, yeah, and maybe like a little bit after that. I think maybe I was dating someone at the time. That kind of faded out and so did that [PrEP use]. I do that, just kind of switch my habits a little bit when things switch up in your life. (Black MSM, age 33)
4.) I stopped using it [PrEP] because I just had kind of like a change of lifestyle, I guess. At first, I was living on the streets and so because I was living on the streets, I kind of was a little bit more encouraged to use PrEP because I was participating in sex work sometimes… So, I wanted to use PrEP for more protection. I was using it for–I don’t know, I want to say probably at least it was going be a week, but I stopped using it, and I still have it. So I think that like I’m going to probably use it in the future, but as of like right now, I’m trying not to really to participate in intercourse at all. (Black MSM, age 23)
5.) There was a good amount of time that I didn’t have it and then I think I got back on it, but then I got in a relationship. At the time, I didn’t really need it because I was in a relationship, so I discontinued. (Latina TW, age 21)
6.) I was starting to have sex with one person who was also on PrEP and we decided that we were going to have an exclusive relationship. So that means no sex outside. It’s just basically between us and we made the–we did get tested and then we stopped PrEP. (Latino MSM, age 34)

### Structural or logistical barriers

Participants noted several structural or logistical barriers that led to PrEP discontinuation. This commonly included a lapse or loss of health insurance experienced after relocating to a new city or beginning a new job, resulting in higher out-of-pocket costs to continue with a PrEP prescription (Table 3, Quotes 1–3). Participants also expressed a general frustration when having to enroll or re-enroll in either private- or publicly-funded health insurance programs, prompting some to change to other methods of HIV prevention altogether (e.g., using condoms consistently; Quote 3). Others described the long distances to pick up prescription refills or attend routine follow-up PrEP medical visits as reasons for stopping (Quote 4).

**Table 3:** Structural or logistical barriers.

1.) In the beginning I had my insurance, but then it expired. So I didn’t take it for three months. (Latina TW, age 23)
2.) The reason I’m not on PrEP anymore is because I switched jobs. So, I had an insurance lapse. I went into the pharmacy and they’re like, “Oh, you owe us $1,200 for your PrEP.” I was like, what? So I stopped taking–or I stopped refilling them for three months and then I got my new insurance from my current provider, but I haven’t gone back to get a new prescription. (Latino MSM, age 23)
3.) When my insurance changed when I got a different job, the insurance provider didn’t offer it as preventative medicine and so the pills were a hundred and something bucks… I was just like, “That’s out.” So, I went from doing it for free then to having to pay a hundred dollars. It’s like, “I don’t know. I’ll just wear condoms.” That was kind of the reason why I stopped… When I got let go from my job, I wanted to start back again, but dealing with the whole medical thing is a pain in my neck. You have to do a lot of paperwork and put out your whole life just to get the runaround for a long time before you get services, and I don’t have time like that. (Black MSM, age 29)
4.) I was tired of going from there [blinded] all the way here to Hollywood and pick up my pills and go back all the way back to my house. It was a big ol' trip and I was tired of doing that. (Latino MSM, age 25)

### Experienced or anticipated medication side effects

The experience or anticipation of medication side effects associated with the use of Truvada for PrEP also emerged as an additional barrier to consistent use. In most cases, side effects manifested in very overt ways (e.g., experiencing digestive or other gastrointestinal issues, elevated creatinine levels), and were alarming enough for participants to cease using the medication (Table 4, Quotes 1–3, 6). Others simply feared that continued use of the medication would have adverse long-term effects on their health (e.g., liver damage) and therefore chose to discontinue (Quote 4).

**Table 4:** Experienced and anticipated medication side effects.

1.) The only thing I can say is that I got sick off of it. I think it’s extremely helpful if your body can withstand the medication, but if you’re body’s not going to keep it, then you can’t take it. (Black MSM, age 26)
2.) I don’t know if it’s related to PrEP or not, but I’ve been having a lot of digestive problems. So, I just want to be off the medication and try to figure that out before I can get back on it. (Latino MSM, age 27)
3.) The last time I was here, they told me my creatinine number and it scared me a little bit. That’s always been an issue with me being on PrEP is that my creatinine level is always higher than normal. The last time, it went even higher than it had been previously. That’s why I went off of it. (Black MSM, age 43)
4.) I have my reservations on taking it. My liver is not so good. So I tend to think that it’s probably going to damage it more, eventually, like, in months or a year or something… One of my friends just tell me not to–because of my liver, not to take it. (Latino MSM, age 40)
***Interactions between PrEP and feminizing hormones***
5.) I was taking it, but I was having issues taking it, and plus I was taking a lot of other medications and stuff. So I ended up stop taking it. (Black TW, age 29)
6.) I got nauseous to be honest in the beginning. I don’t know what it was, but I felt a little nauseous and I am a hypochondriac just a little bit–to some degree. Anything that happens to me, I think it’s a derivative of something I’m taking. I’m also on hormones, so I have all this stuff going into my body and I’m not the tender age of twenty or thirty anymore. I’m just skeptical about all the stuff that I’m putting into my body. At the same time, I want to live a vibrant, vivacious, stunning life and I don’t want anything I don’t have. Not today. (Black TW, age 48)
7.) I would drink sometimes and I just don’t want to mix. I [also] take hormone pills. Even though I take shots every two weeks, but I just didn’t want too much drugs. (Latina TW, age 24)

#### Interactions between PrEP and feminizing hormones

BLTW described experiences of temporary PrEP discontinuation related to a fear of consuming multiple medications (e.g., feminizing hormones) in addition to PrEP and not fully understanding the interactions between them (Quotes 5–7). However, these women ultimately saw the utility of PrEP in improving their quality of life and/or lessening their risk of HIV infection, and, therefore, chose to re-initiate PrEP (Quote 6).

### Challenges with medication adherence

An additional theme emerged related to challenges participants experienced with medication adherence. Because participants understood the significance of adherence to the effective use of PrEP, they viewed their inconsistent use of the medication as a justifiable reason for stopping (Table 5, Quote1). Others recognized their inability to remain adherent, in general, as shaping their decision to discontinue PrEP (Quote 2). In place of PrEP, many of these participants either remained exclusive to their primary partner or refrained from having sex altogether (i.e., not “participating in intercourse”). One participant who re-initiated PrEP made an ardent attempt to improve her adherence after being encouraged by her provider to do so, but still struggled with remembering to take her medication (Quote 3).

**Table 5:** Understanding the importance of adherence.

1.) My adherence was just not that great, but it wasn’t like I completely stopped. I just kept telling myself to keep trying and trying and trying. Then, just like I said, about a month ago–maybe less, like three weeks–I decided that I can’t be doing on and off, on and off. So I decided to stop completely until further notice. (Latino MSM, age 27)
2.) I’m always stopping and using it. I don't think that I've gone through a whole two months or a month using PrEP” (Black MSM, age 23, 25 months on PrEP)
3.) I have not been consistent. I had to go back and my doctor said, “Please be consistent.” So I’ve been practicing being consistent for the last month or so… I’m supposed to take it every single night. I’m going to be honest, some nights I say, “Ooh, I’m going to take my pill!” And I forget because I lay down. (Black TW, age 48)

## Discussion

In this study, we identified structural, logistical, and individual-level factors related to PrEP discontinuation among BLMSM and BLTW. Structural/logistical factors were beyond the control of the individual and included barriers to access (e.g., costs, loss of insurance, difficulty navigating complex medical systems). Individual factors involved a conscious decision on the part of users to discontinue PrEP (e.g., recognizing their inability to adhere to the medication, no longer engaging in HIV sexual risk behaviors). In addition, participants also discontinued the medication after experiencing or anticipating side effects, or, in the case of transgender women, expressing a fear about managing multiple medications. Helping users remain adherent to PrEP is an important piece of the PrEP care continuum and will help ensure that users remain HIV uninfected while on the medication. As PrEP is recommended as an essential strategy for the “Ending the HIV Epidemic: A Plan for America” initiative [9], PrEP programs and providers will need to recognize that some BLMSM and BLTW PrEP users may require additional support, beyond medical services, to help them remain persistent with their PrEP use.

In this study, participants cited structural and logistic issues such as changes or a lapse in health insurance, high cost of medication co-pays, and difficulties navigating a complex medical system as reasons for discontinuing PrEP. These findings align with other studies with racial/ethnic, sexual, and gender minorities [11, 17, 19, 20]. As we consider ways to reduce HIV infections among highly impacted minority populations, it is important to address these structural barriers to PrEP continuation from a health equity perspective, as many of these factors are rooted in the social determinants of health experienced by these populations [21, 22]. The establishment of universal health care coverage could greatly improve access and persistent use of PrEP among highly vulnerable populations. Until then, low-income, marginally employed, un- or under-insured individuals will need to rely on available private and public, local, state, and national resources for PrEP access (e.g., pharmaceutical company medication assistance programs, insurance co-pay assistance, publicly funded insurance programs).

BLMSM and BLTW participants also described a host of individual factors that led to PrEP discontinuation. Primary among them were challenges with medication adherence. Struggles with daily adherence to PrEP is something common in many populations, particularly racial/ethnic, sexual, and gender minority populations [14, 23, 24]. Intervention trials are underway to test simple mobile technology products (e.g., text messaging services, mobile applications) to support PrEP adherence [25–27]. In addition, there are ongoing investigations of alternative dosing strategies (e.g., intermittent dosing) that may be more acceptable and manageable for persons unable to adhere to daily dosing [28, 29]. A long-acting injectable version of PrEP (Cabotegravir) is also awaiting approval by the US Food and Drug Administration and may prove useful for individuals who experience difficulty taking a daily medication [32]. There are also simple tools that PrEP users can use to facilitate daily use (e.g., mobile phone reminders, pill cases). It is important that PrEP providers, and their teams, identify other potential strategies or dosing methods to address adherence issues, particularly among highly impacted populations.

Some participants in the study made a conscious decision to stop PrEP based on changes in their sexual behaviors or life circumstances, such as entering into a monogamous relationship or choosing not to engage in high risk sexual behaviors (e.g., condomless sex). While PrEP is an important HIV prevention tool, it is also important to respect the autonomy of an individual to discontinue the medication following a decrease in sexual risk taking. This behavior change should be augmented with supportive counseling around alternative strategies or methods to remain HIV negative (e.g., reducing number of sexual partners, strategic positioning, consistent condom use).

An additional individual-level factor that caused some BLMSM and BLTW to stop PrEP was their experiences with medication side effects. These included both short- and long-term side effects as well as interactions between PrEP and other medications, particularly feminizing hormones used by transgender women. The fear of interactions between hormones and PrEP has been reported as a reason why some TW do not initiate PrEP [30]. Furthermore, one study indicates that feminizing hormones might impact the efficacy of Truvada for PrEP among TW [31]. Adverse effects are a logical reason for discontinuing PrEP; however, it is important that users struggling with side effects or who have concerns about drug-drug interactions have the opportunity to discuss these issues with a medical provider or support staff to explore other ways of taking the medication that may lessen side effects (e.g., taking medication with a meal, changing the time of day medication is taken). In addition, with the FDA approval of Descovy [8], BLMSM and BLTW now have the option to initiate an alternative PrEP medication (e.g., Descovy) should they experience side effects with Truvada.

### A need for PrEP support services

Based on the reasons for discontinuing PrEP identified in the present study, and the challenges to PrEP persistence and retention noted in other studies [11, 19, 20, 24, 32], we believe there is a critical need for support services for BLMSM and BLTW to remain persistent with their PrEP regimen. These services should focus on the behaviors associated with effectively managing a PrEP prescription (i.e., adherence, persistent use, and attendance at PrEP medical visits) to ensure the optimal performance of PrEP, and could take the form of either PrEP case management or expanded PrEP navigation services, possibly co-located with a PrEP medical provider. In prior research, case management has proven effective in retaining people living with HIV in medical care and achieving improved medication adherence [33–35]. In a few studies, PrEP navigation services have also shown to support continued PrEP engagement [36–38]. PrEP support services will be particularly important during the first 12 months of PrEP use, as prior research has indicated that many BLMSM and BLTW discontinue the medication within 6 to 12 months after initiation [28, 39].

Expanding PrEP navigation services or developing a PrEP case management program are two potential options to support PrEP persistence. At present, many existing PrEP navigation services support individuals in initiating PrEP (e.g., providing information on accessing medication assistance programs, answering questions about using PrEP, disseminating information on where to access PrEP, accompanying individuals to initial PrEP medical visit). This service should be expanded to include PrEP retention efforts after initiation, as some of the barriers or challenges to starting PrEP are the same for remaining on PrEP (e.g., lack or lapse of insurance, cost, side effects) [40–43]. Case management, which has been shown to be effective in managing HIV care, is another option to support PrEP users [44–46]. This could involve regular phone, text, or in-person check-ins with PrEP users to monitor their PrEP care (i.e., attendance at regular medical monitoring visits, completing prescription refills, adherence to and continued use of the medication). PrEP case managers would be available to address issues that may arise in the continued use of PrEP to remain HIV uninfected.

Support services may also take a more proactive and technology-based approach by using simple tools such as text messages to send encouraging and affirming messages about continuing to use PrEP (e.g., “PrEP helps protect your beautiful body,” “Have a fulfilling and healthy sex life, you deserve it!”). Health-related text messaging services can be automated, in-person, unidirectional, or bidirectional [47–49]. Text messaging interventions have been proven effective in supporting medication adherence and retention in medical care among persons living with HIV [47–50], and may therefore be a viable option to support PrEP persistence among PrEP users.

### Limitations

These findings should be interpreted within the context of our study’s limitations. We recruited our sample of BLMSM and BLTW PrEP users in Los Angeles. As such, these insights may not be generalizable to the experiences of BLMSM and BLTW in other locations. In addition, the sample consists exclusively of English-speaking BLMSM and BLTW and may not reflect the experiences of Spanish-speaking MSM and TW PrEP users. Research with monolingual Spanish-speaking PrEP users is needed to assess if reasons for discontinuation differ based on language. Another limitation is that we were unable to conduct follow-up interviews with BLTW and were therefore unable to assess changes in PrEP use overtime.

## Conclusions

PrEP is an important prevention strategy for some BLMSM and BLTW, particularly during periods of greater HIV risk. Understanding the nuances of why PrEP discontinuation occurs in these populations will help inform the development of support services to help these populations remain persistent with PrEP to remain HIV uninfected. Future research may also want to explore the narratives of long-term PrEP users who are able to effectively manage their PrEP use (i.e., remaining adherent, persistent, and retained in PrEP medical care) to identify resiliency factors that may be transferrable for BLMSM and BLTW struggling with using PrEP. In prior work, we identified social support from family, peers, and friends as a resiliency factor that contributed to the continued use of PrEP among BLMSM and BLTW, despite experiences of PrEP-related stigma. We also identified PrEP advocacy as a supportive factor in the continued use of PrEP. Some BLMSM and BLTW who recognized the mental and physical health benefits of their PrEP use emerged as PrEP advocates and routinely provided information to peers about the importance of PrEP in preventing HIV infection, corrected misinformation, or challenged PrEP stigma when it occurred [19–21].

## Supporting information

S1 Appendix Interview guide. (DOCX)
