# Supplementary material for: PrEP discontinuation among Latino/a and Black MSM and transgender women: A need for PrEP support services
Source: PLoS One. 2020 Nov 5;15(11):e0241340. doi: 10.1371/journal.pone.0241340 (PMC7644013; doi:10.1371/journal.pone.0241340)
Supplement: S1 Appendix — (DOCX) [file pone.0241340.s001.docx]

**S1 Appendix**

**Interview Guide**

[Interviewer opening prompt to qualitative questions]

I will be tape recording this part of the interview. As I mentioned before, your name will not be attached to this interview in any way. Instead, we will use PID#_______ from this point forward. We will transcribe this later for our analyses.

I will be asking you questions about how you came to use PrEP for HIV prevention and who you’ve talked to about your PrEP use and how they have reacted when you told them that you are using PrEP. Do you have any questions before we begin?

[Interviewer responds to any questions and then begins interview]

1. How is/was your PrEP being covered or paid for? (e.g., private insurance, through your medical doctor, through a demonstration project or research study)

[This question will help contextualize each participant’s PrEP adoption process].

1. Where are you getting PrEP from?
2. How did you first learn about PrEP?
3. What were your main sources for PrEP information?
4. Were there any persons you talked to about PrEP before deciding to use it?
5. What was the main reason you decided to use PrEP?
6. When did you first start taking PrEP, the approximate date of when you started?
7. What were some of the challenges you faced in accessing PrEP?
8. Did you ever start or stop using PrEP?
9. If you did stop, why did you stop?
10. When did you stop using PrEP? Approximately.
11. How long did you stop using PrEP?
12. Did you tell your doctor that you stopped?
13. Why did you start again?
